# Supplementary material for: Human Trabecular Meshwork (HTM) Cells Treated with TGF-β2 or Dexamethasone Respond to Compression Stress in Different Manners
Source: Biomedicines. 2022 Jun 6;10(6):1338. doi: 10.3390/biomedicines10061338 (PMC9219943; doi:10.3390/biomedicines10061338)
Supplement: Supplementary file 1 [file biomedicines-10-01338-s001.zip › biomedicines-1736965-supplementary (1).pdf]

Supplemental Table S1. Sequences of primers of qPCR.

		Sequence	Exon Location	RefSeq Number	Product Length (bp)
human RPLP0[*1]	Probe<br>Primer2<br>Primer1	5'-/56-FAM/CCCTGTCTT/ZEN/CCCTGGGCATCAC/3IABkFQ/-3'<br>5'-TCGTCTTTAAACCCTGCGTG-3'<br>5'-TGTCTGCTCCCACAATGAAAC-3'	2-3	NM_001002	143
human COL1A1 [*1]	Probe<br>Primer2<br>Primer1	5'-/56-FAM/TCGAGGGCC/ZEN/AAGACGAAGACATC/3IABkFQ/-3'<br>5'-GACATGTTCAGCTTTGTGGAC-3'<br>5'-TTCTGTACGCAGGTGATTGG-3'	1-2	NM_000088	131
human COL4A1 [*1]	Probe<br>Primer2<br>Primer1	5′-/56-FAM/TCATACAGA/ZEN/CTTGGCAGCGGCT/3IABkFQ/-3′<br>5′-AGAGAGGAGCGAGATGTTCA-3′<br>5′-TGAGTCAGGCTTCATTATGTTCT-3′	51-52	NM_001845	117
human COL6A1 [*1]	Probe<br>Primer2<br>Primer1	5′-/56-FAM/CAGGTTTCG/ZEN/GTCACAGCGGTAGT/3IABkFQ/-3′<br>5'-CCTCGTGGACAAAGTCAAGT-3'<br>5'-GTGAGGCCTTGGATGATCTC-3'	2-3	NM_001848	141
human FN1[*1]	Probe<br>Primer2<br>Primer1	5′-/56-FAM/TACAGCTTA/ZEN/TTCTCCCTCGCCCAG/3IABkFQ/-3′<br>5'-CGTCCTAAAGACTCCATGATCTG-3'<br>5'-ACCAATCTTGTAGGACTGACC-3'	3-4	NM_212482	114
human αSMA[*1]	Probe<br>Primer2<br>Primer1	5'-/56-FAM/AGACCCTGT/ZEN/TCCAGCCATCCTTC/3IABkFQ/-3'<br>5'-AGAGTTACGAGTTGCCTGATG-3'<br>5'-CTGTTGTAGGTGGTTTCATGGA-3'	8-9	NM_001613	130
human Grp78[*2]	Forward<br>Reverse	5'-CATCACGCCGTCCTATGTCG-3'<br>5'-CGTCAAAGACCGTGTTCTCG-3'		NM_005347	104
human GRP94[*2]	Forward<br>Reverse	5'-CTGGGACTGGGAACTTATGAATG-3'<br>5'-TCCATATTCGTCAAACAGACCAC-3'		NM_003299	217
human XBP[*2]	Forward<br>Reverse	5'-AGTAGCAGCTCAGACTGCCA-3'<br>5'-CCTGGTTCTCAACTACAAGGC-3'		NM_005080	152
human sXBP[*2]	Forward<br>Reverse	5'-GGTCTGCTGAGTCCGCAGCAGG-3'<br>5'-GGGCTTGGTATATATGTGG-3'		AB076384	313
Human IL1B[*1]	Probe<br>Primer2<br>Primer1	5'-/56-FAM/AGAAGTACC/ZEN/TGAGCTCGCCAGTGA/3IABkFQ/-3'<br>5'-CAGCCAATCTTCATTGCTCAAG-3'<br>5'-GAACAAGTCATCCTCATTGCC-3'	1-3	NM_000576	105
Human IL6[*1]	Probe<br>Primer2<br>Primer1	5'- /56-FAM/CAACCACAA/ZEN/ATGCCAGCCTGCT/3IABkFQ/-3'<br>5'-GCAGATGAGTACAAAAGTCCTGA-3'<br>5'-TTCTGTGCCTGCAGCTTC-3'	4-5	NM_000600	120

*1 Taqman probes (IDT, Coralville, IA, USA). *2 SYBR probes (IDT, Coralville, IA, USA).

Supplemental Table S2. Protocol for Tagman and SYBR qPCR.

Taqman: Cycling Protocol

Step	Cycles	Temperature (℃)	Cycling (min: sec)
Polymerase activation	1	95	3:00
Amplification	35-45		
Denaturation		95	0:05
Annealing/Extension		60	0:30

SYBR: Cycling Protocol

Step	Cycles	Temperature (℃)	Duration (min: sec)
UDG activation	Hold	50	2:00
Dual-Lock™ DNA polymerase	Hold	95	2:00
Denature	40	95	0:01
Anneal/extend	40	60	0:30

SYBR: Melt curve stage

Step	Ramp rate	Temperature (℃)	Time (min: sec)
1	1.6℃/second	95	0:15
2	1.6℃/second	60	1:00
3*1	0.15℃/second	95	0:15

*1 Dissociation

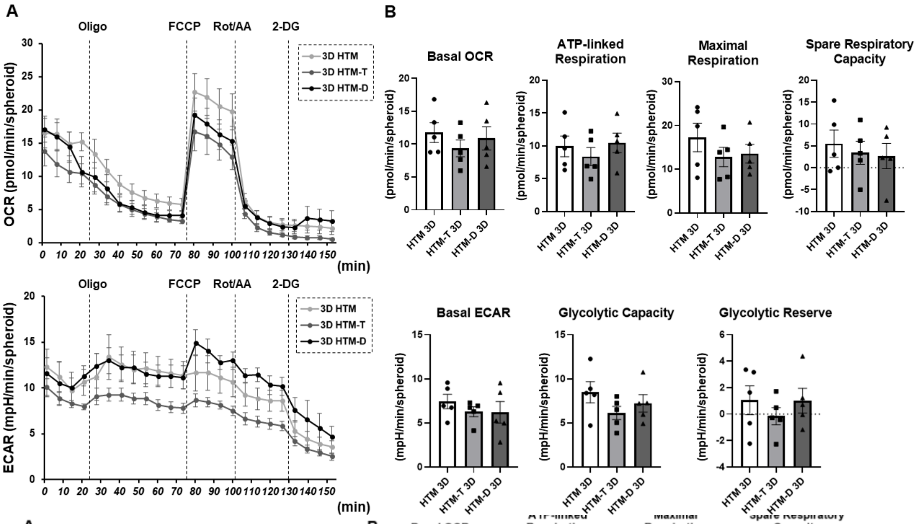
A
Oligo
FCCP
Rot/AA
2-DG
3D HTM
3D HTM-T
3D HTM-D
OCR (pmol/min/spheroid)
ECAR (mpH/min/spheroid)
(min)
B
Basal OCR
ATP-linked Respiration
Maximal Respiration
Spare Respiratory Capacity
Basal ECAR
Glycolytic Capacity
Glycolytic Reserve
(pmol/min/spheroid)
(mpH/min/spheroid)
HTM 3D
HTM-T 3D
HTM-D 3D

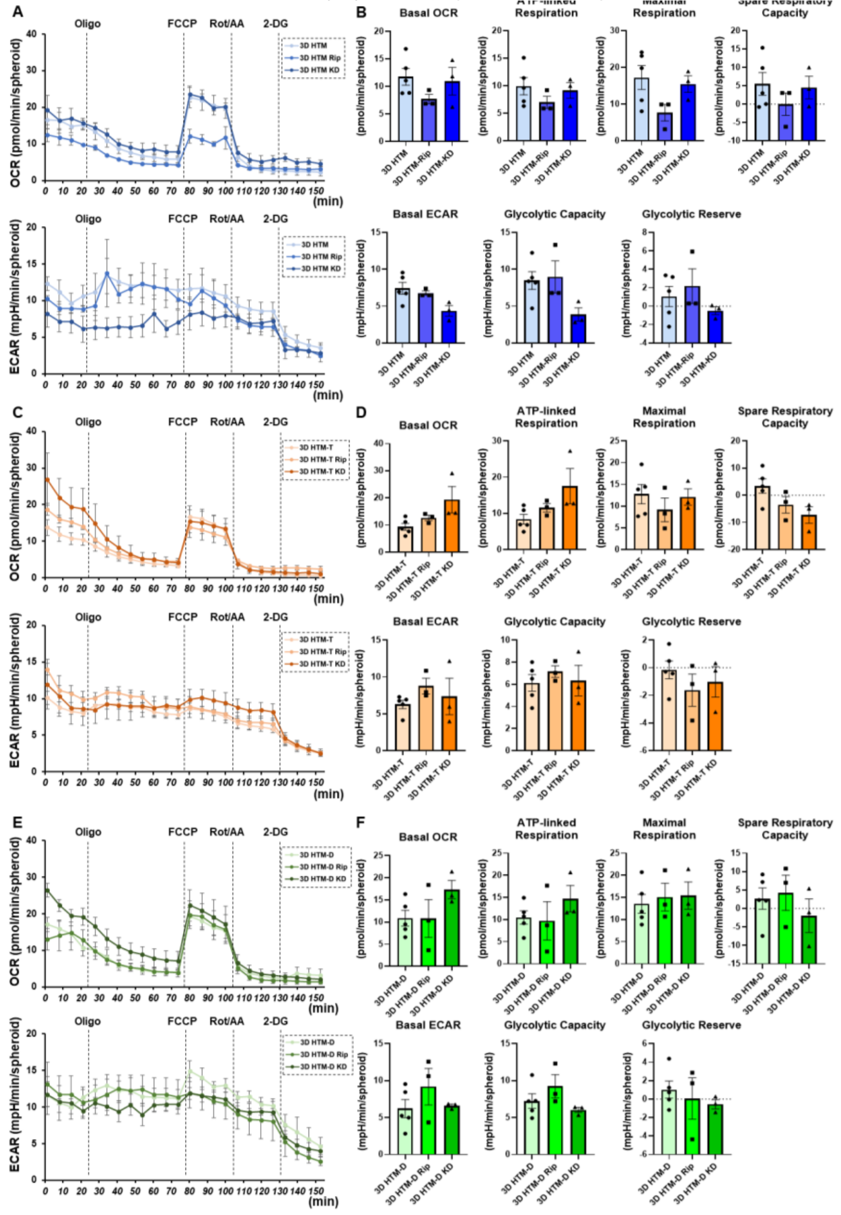
A
Oligo
FCCP
Rot/AA
2-DG
3D HTM
3D HTM Rip
3D HTM KD
OCR (pmol/min/spheroid)
ECAR (mpH/min/spheroid)
(min)
B
Basal OCR
ATP-linked Respiration
Maximal Respiration
Spare Respiratory Capacity
Basal ECAR
Glycolytic Capacity
Glycolytic Reserve
(pmol/min/spheroid)
(mpH/min/spheroid)
3D HTM
3D HTM-Rip
3D HTM-KD
C
3D HTM-T
3D HTM-T Rip
3D HTM-T KD
D
E
3D HTM-D
3D HTM-D Rip
3D HTM-D KD
F

Supplemental Figure S1. Measurement of mitochondrial and glycolytic functions of TGF-β2 or DEX treated or untreated 3D cultured HTM cells in the absence or presence of ROCK inhibitors (ROCK-is).

Among following 4 conditions; 1) 3D HTM spheroids (HTM) and those treated by 5 ng/ml TGF-β2 (HTM-T) or 250 nM DEX (HTM-D), 2) untreated 3D HTM spheroids treated without (HTM) or with 10 μM pan-ROCK-i, ripasudil (HTM Rip) or ROCK2-i, KD025 (HTM KD) for 24 hours, 3) 5 ng/ml TGF-β2 (TGF) treated 2D cultured HTM cells treated without (HTM-T) or with 10 μM pan-ROCK-i, ripasudil (HTM-T Rip) or ROCK2-i, KD025 (HTM-T KD) for 24 hours, and 4) 250 nM DEX treated 3D HTM spheroids treated without (HTM-D) or with 10 μM pan-ROCK-i, ripasudil (HTM-D Rip) or ROCK2-i, KD025 (HTM-D KD) for 24 hours, 3D HTM spheroids were subjected to mitochondrial and glycolysis function analyses using a Seahorse XFe96 Bioanalyzer. Measurements of oxygen consumption rate (OCR) and extracellular acidification rate (ECAR) before drug injections (at baseline) were calculated as 100 % and their changes were determined by the following injections: (i) oligomycin (a complex V inhibitor), (ii) FCCP (a protonphore), (iii) rotenone/antimycin (complex I/III inhibitors), and (iv) 2-DG (a hexokinase inhibitor). (A) Measurements of OCR and ECAR. (B) Plots for subcomponents of OCR (basal, ATP-linked Respiration, Maximum Respiration and Spare Respiratory Capacity) and ECAR (basal, Glycolytic Capacity and Glycolytic Reserve). ATP-linked Respiration defined as average of OCR in the presence oligomycin. Maximum Respiration defined as average of OCR in the presence FCCP. Spare respiratory reserve defined as the difference between an average of OCR in the presence FCCP and an average of OCR at baseline. Glycolytic reserve defined as the difference between the final measurement of ECAR in the presence of oligomycin and an average of ECAR. Data are presented as the mean ± the standard error of the mean (SEM).

COL1

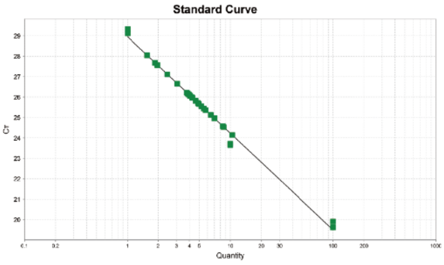

GRP78

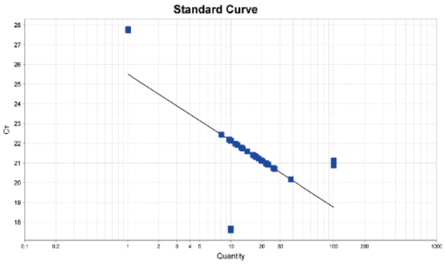

COL4

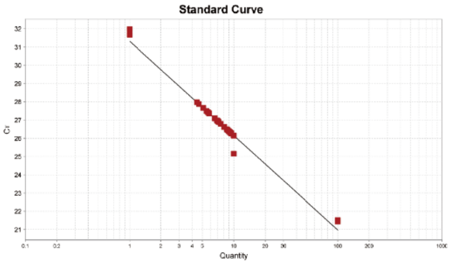

GRP94

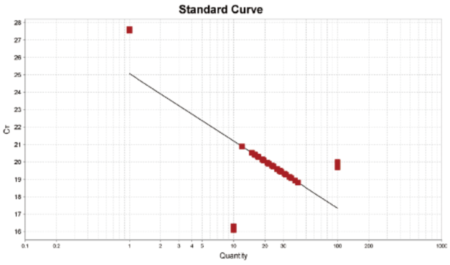

COL6

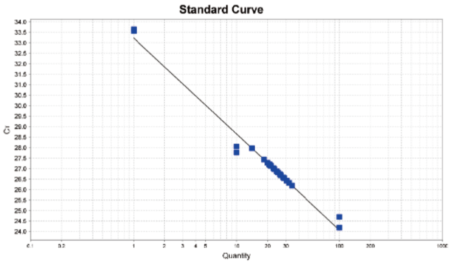

sXBP

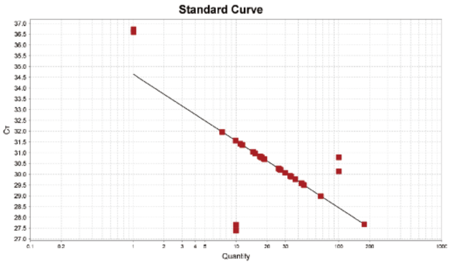

FN

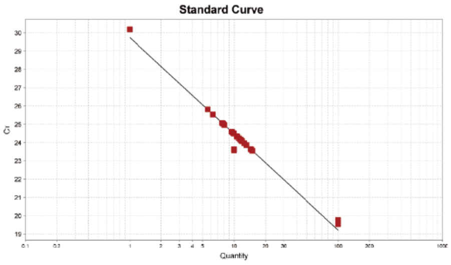

XBP

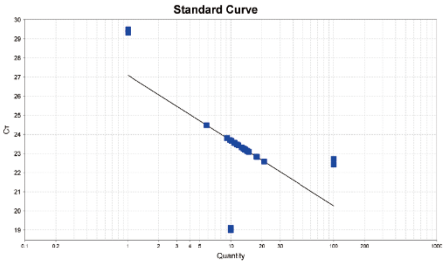

SMA

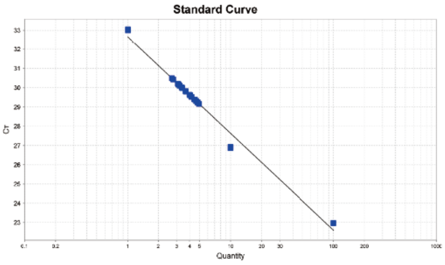

IL6

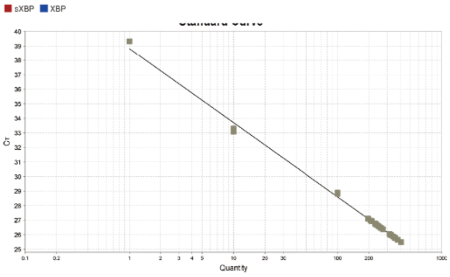

IL1B

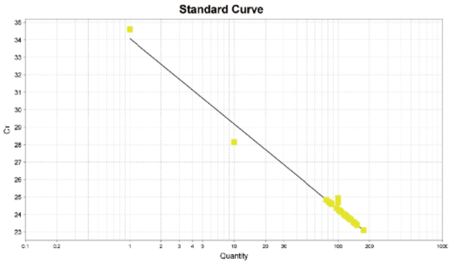

Supplemental Figure S2. The standard curves of each genes of the qPCR analysis.

The standard curves of each genes of the qPCR analysis of non-treated control are shown. Similar to these, the linearity analyses data of all genes were also confirmed in terms of other experimental conditions, DEX and TGF-β2 (data not shown).

Supplemental Method. Measurement of mitochondrial and glycolytic functions of the 3D HTM spheroids.

Oxygen consumption rate (OCR) and extracellular acidification rate (ECAR) of 5 ng/ml TGF-β or 250 nM DEX untreated or threated 3D HTM spheroids and those treated with 10 μM pan-ROCK-i, ripasudil and ROCK2-i, KD025 for 24 hours were each measured using Seahorse XFe96 Bioanalyzer as described in the Method using 2D HTM cells. In brief, 3D spheroids were washed in PBS twice and the number of five spheroids were then transferred into a well of a XFe96 Spheroid Microplate (Agilent Technologies, # 102978-100) containing 180 μL of the assay buffer. The assay plates were incubated in CO_2-free incubator at 37 °C for 1 hour prior to the measurement. OCR and ECAR were measured in the Seahorse XFe96 Bioanalyzer under 3 mins mix and 3 mins measure protocols at baseline and following injections of oligomycin (final concentration: 2.0 μM), carbonyl cyanide p-trifluoromethoxyphenylhydrazone (FCCP, final concentration: 5.0 μM), a mixture of rotenone/antimycin A (final concentration: 1.0 μM), and 2-deoxyglucose (2-DG, final concentration: 10 mM). Considering the difference in the effect of drug injection between 2D and 3D conditions, 3 cycles in each measurement were set in the 2D cells, and 8 cycles for a measurement with oligomycin and 4 cycles for other measurements were set in the 3D spheroids.
